# Balancing on a Slackline: 8-Year-Olds vs. Adults

**DOI:** 10.3389/fpsyg.2013.00208

**Published:** 2013-04-22

**Authors:** Andrea Melanie Schärli, Melanie Keller, Silvio Lorenzetti, Kurt Murer, Rolf van de Langenberg

**Affiliations:** ^1^Institute of Human Movement Sciences and Sport, ETH ZurichZurich, Switzerland; ^2^Institute of Sport Science, University of BernBern, Switzerland; ^3^Institute of Biomechanics, ETH ZurichZurich, Switzerland

**Keywords:** postural control, stability, head, gaze, trunk, balance, slackline, children

## Abstract

Children are less stable than adults during static upright stance. We investigated whether the same holds true for a task that was novel for both children and adults and highly dynamic: single-legged stance on a slackline. We compared 8-year-olds with young adults and assessed the following outcome measures: time on the slackline, stability on the slackline (calculated from slackline reaction force), gaze movement, head-in-space rotation and translation, trunk-in-space rotation, and head-on-trunk rotation. Eight-year-olds fell off the slackline quicker and were generally less stable on the slackline than adults. Eight-year-olds also showed more head-in-space rotation and translation, and more gaze variability around a visual anchor point they were instructed to fixate. Trunk-in-space and head-on-trunk rotations did not differ between groups. The results imply that the lower postural stability of 8-year-olds compared to adults – as found in simple upright stance – holds true for dynamic, novel tasks in which adults lack the advantage of more practice. They also suggest that the lack of head and gaze stability constitutes an important limiting factor in children’s ability to master such tasks.

## Introduction

Many studies have investigated the development of postural control in children during quiet upright stance. Children are less stable than adults and postural stability (PS) improves as a function of age (e.g., Shumway-Cook and Woollacott, 1985; Cherng et al., 2001; Rival et al., 2005; Ionescu et al., 2006; Steindl et al., 2006; Cumberworth et al., 2007; Ferber-Viart et al., 2007; Hsu et al., 2009; Cuisinier et al., 2011; Schärli et al., 2012) and motor competence of children (e.g., Roncesvalles et al., 2001). Likewise, it has been shown that the decreased PS in children as compared to adults persists or even becomes more pronounced under conditions of an unstable support surface or sensory conflict (Lee and Aronson, 1974; Peterka and Black, 1990; Foudriat et al., 1993; Berger et al., 1995; Hirabayashi and Iwasaki, 1995; Golomer et al., 1997; Ionescu et al., 2006; Steindl et al., 2006; Cumberworth et al., 2007; Mallau et al., 2010; Fujiwara et al., 2011). Moreover, children were found to adapt less well to changes in the oscillation frequency of a moving support surface than did adults (Berger et al., 1995).

Most studies assessing dynamic balance in children (e.g., Peterka and Black, 1990; Foudriat et al., 1993; Hirabayashi and Iwasaki, 1995; Ionescu et al., 2006; Steindl et al., 2006; Cumberworth et al., 2007) used the widely accepted Computerized Dynamic Posturography (CDP) test series called the sensory organization test (SOT) developed by Nashner (1997). In this test series, three conditions with unstable surface are included. Other studies used a treadmill moving sinusoidally forward-backward in relation to the subject at different frequencies (Berger et al., 1995; Mallau et al., 2010), an oscillator fixed to a force platform (Fujiwara et al., 2011) or a stabilometer platform (Golomer et al., 1997) to study children’s dynamic balance.

The reasons offered for children’s lower PS in static and the above described dynamic situations have been threefold. First, it may be due to an immaturity of the involved sensory systems (i.e., visual, vestibular, somatosensory). In line with this idea, Cumberworth et al. (2007), Steindl et al. (2006), and Hirabayashi and Iwasaki (1995) have shown – by means of CDP – that proprioceptive function in stance stability is adult-like between 3 and 4 years of age, whereas the visual and vestibular systems reach adult-like levels only at 15–16 years of age. A second explanation, offered by Woollacott et al. (1987), Peterson et al. (2006), and Cuisinier et al. (2011), is that it is the integration between sensory systems, rather than the sensory systems themselves, that is underdeveloped in children. Supportive of this assumption are studies that show that children have more problems than adults when they have to balance under conflicting sensory conditions (e.g., Peterka and Black, 1990; Cherng et al., 2001; Hsu et al., 2009). Third, the lower PS in children might be due to a lower degree of intersegmental coordination reflected in a hampered head stabilization, as suggested by Assaiante and Amblard (1995), Berger et al. (1995), Mallau et al. (2010), and Schärli et al. (2012). Head stabilization in space is not only important in stationary tasks, but also in locomotor tasks such as walking, running, and hopping on one foot (Pozzo et al., 1990). An unstable head may lead to an impaired function of the visual and vestibular systems and hence impair postural control. Moreover, movement of the head may be a direct, mechanical source of postural instability during stance and locomotion. The difference in head stabilization between young children and adults, as observed during quiet stance, may be further amplified in a challenging balance situation, where stabilizing the head is more difficult.

The above described studies on postural control development under dynamic conditions did not assess such challenging balance situations. In the present study, it was our aim to compare children and adults in their ability to balance on a slackline, which presents a highly challenging and dynamic balancing task that is unfamiliar to both children and adults. In such a situation as compared to static and easier dynamic balancing tasks investigated so far, head stabilization in space is sufficiently challenged and might thus constitute a limiting factor for balance success.

As described by Keller et al. (2012), the first trials on the slackline result in an uncontrollable lateral swinging of the supporting leg and line and an almost immediate loss of balance. Only few attempts on the slackline are required, however, to arrest the leg oscillations and maintain standing balance on the line for longer. We thus set out to compare 8-year-olds’ (the earliest age at which balancing on a slackline becomes feasible, as suggested by an experienced slackline instructor) and adults’ postural strategies while standing on a slackline with one leg.

As a first objective measure of balance success, we measured balance duration. Second, we looked into slackline reaction forces and displacements as a measure of PS. Third, we specifically focused on the role of head-in-space stability, which has been said to play a crucial role in postural control development (Assaiante and Amblard, 1995; Berger et al., 1995; Mallau et al., 2010; Schärli et al., 2012) yet has scarcely been investigated in postural control studies involving children.

Besides aspects of head stability we assessed gaze behavior during balancing as a fourth measure. As with head stability, gaze stability (i.e., the maintenance of a stable visual anchor point) might also be important in challenging balance situations. It has been shown that subjects sway less during quiet stance with target fixation than without fixation and that stability gains are larger when the target that is fixated is closer (Paulus et al., 1989; Stoffregen et al., 2000; Kapoula and Le, 2006). Moreover, Schärli et al. (2012) found that shifting gaze during quiet stance degrades PS more in children than in adults. The latter finding might be due to an inefficient gaze strategy: children move their heads more than adults when shifting gaze, which impedes PS. If children’s PS decreases during gaze shifts in quiet stance, gaze movements (GM) during a dynamic task such as balancing on a slackline might have an even larger negative impact on PS. Therefore, we investigated whether children and adults differ in their ability to fixate their gaze during balancing on the slackline and how this ability relates to balancing achievement.

Results of Cumberworth et al. (2007), Steindl et al. (2006), and Hirabayashi and Iwasaki (1995) suggest that proprioceptive function is adult-like between 3 and 4 years of age. As a fifth and final outcome measure, we adopted a proprioception test to verify this suggestion. As proprioception arguably plays a central role in standing balance, the test hence allows us to assess whether proprioceptive immaturity could explain differences in balance success between 8-year-olds and adults.

Finally, because balancing on the slackline was a novel, highly unfamiliar balance task for both children and adults, we were able to compare the quality of the postural control system as such, not confounded with differences in task exposure. Put differently, although in most balancing tasks adults will have an “unfair” advantage over children because adults will simply have had more experience with them; this is not the case for balancing on a slackline. Furthermore, the present study is unique in terms of the measurement of three-dimensional head-in-space rotations and gaze behavior during balancing. Most studies in postural control development exclusively assess center-of-pressure (COP), without taking into account differences in head stabilization and/or gaze behavior. Hence, the current study is not only novel in its experimental task (and the particular measure of PS in this task), but also in its outcome measures and the insights between-group comparisons on these outcome measures provide.

We hypothesized that children would balance more poorly on the slackline than adults due to less effective postural control. Furthermore, we hypothesized that head-in-space stability would be an important factor underlying children’s less effective postural control.

## Materials and Methods

### Participants

A total of 31 healthy participants took part in this study: one group of children (aged 7.8 ± 0.6, *n* = 20) and one group of young adults (aged 24.6 ± 2.8, *n* = 10). The rationale behind choosing a larger group of 8-year-olds was that children are more variable in their level of postural development than adults so that a larger variability in balance-related outcome measures could be expected. To account for the larger variability in this group, that is, to obtain a better estimate of the outcome measures at the population level, a larger sample was taken. All participants took part voluntarily and had never balanced on a slackline before the start of the study. Approval for the study was given by the local Ethics Committee. Adult participants gave written informed consent. The children gave verbal informed consent and their parents gave written informed consent. Anthropometric data of all participants are presented in Table 1.

**Table 1:** **Anthropometric data of all participants**.

Group	Children (*n* = 21)	Adults (*n* = 10)
Height (m)	1.24 ± 5.9	1.70 ± 6.1
Weight (kg)	23.4 ± 2.9	62.3 ± 7.8
Gender (m/f)	10/11	3/7

### Apparatus

#### Slackline

**Figure 1:**
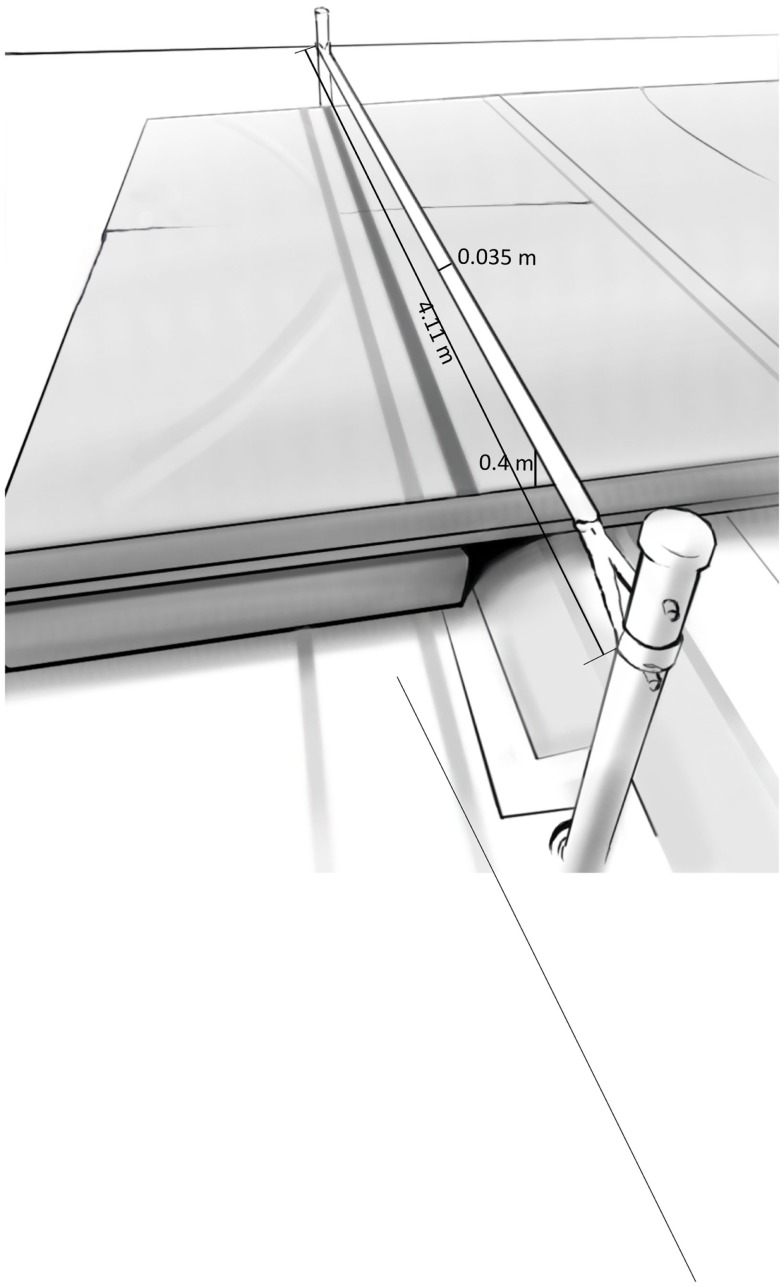
**Experimental setup of the slackline for single-legged balance measurements with gaze fixed on the smiley**.

Slacklining is a balance sport similar to tight-rope walking whereby a nylon webbing is stretched tight between two anchor points. The difference with tightrope walking lays in its dynamic nature. The line is not held rigidly taut and therefore allows for bouncing and stretching. Moreover, the slackline itself is a flat belt rather than a cylindrical rope, which somewhat stabilizes the foot in the roll direction. The slackline used in the present study (Slacktivity GmbH, Wartau, Switzerland) was fixed between two poles that stood 4.11 m apart (see Figure 1). The height of the slackline above the ground was 0.4 m, its width 0.035 m. Tension of the slackline was adjusted to each participant’s body weight in order to reach a vertical displacement of the slackline between 0.18 and 0.2 m for all participants. The center of the slackline was marked and used as the starting point for each balance measurement. Mats covered the ground underneath the slackline ensuring soft landings.

#### Force sensors

Two force sensors (type KM30z, ME_Messsysteme GmbH, Hennigsdorf, Germany) were each attached between the fixed-point and the slackline on both sides. They had a measurement range up to 50 kN and were sampled at 100 Hz.

#### Motion capture

**Figure 2:**
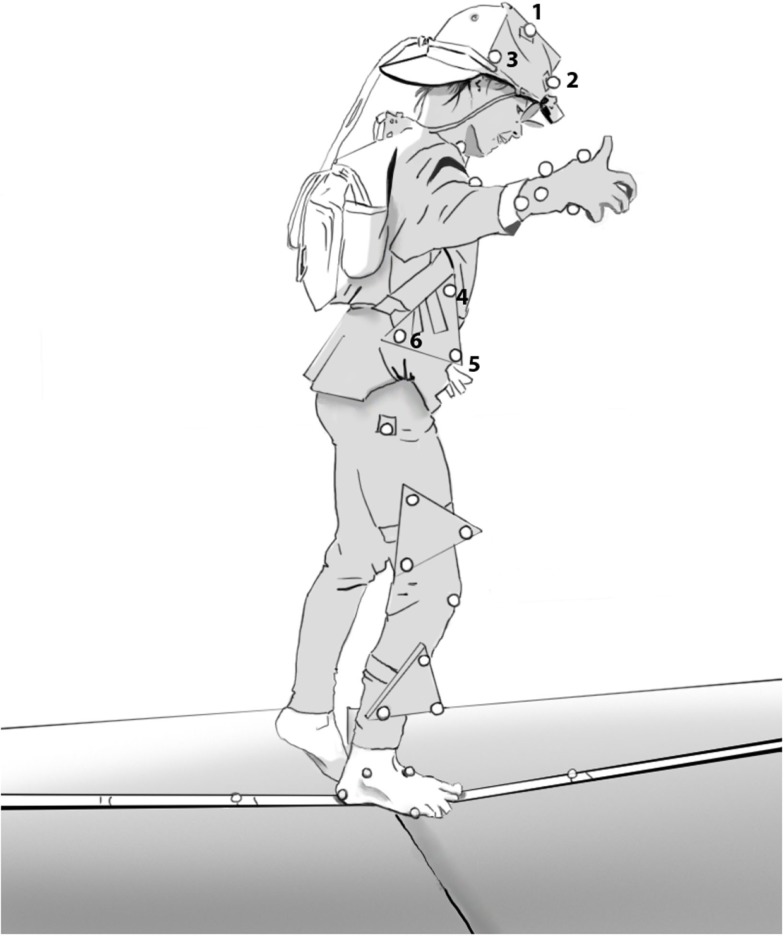
**Marker placement**. Head markers top (1), front (2), back (3) and trunk markers top (4), front (5), back (6).

Vicon Motus 9.2 is a video-based 3D motion analysis system. Reflecting markers of 9 mm diameter were placed on the standing foot and on the slackline; markers of 16 mm diameter were placed on the arm, head (markers 1–3, fixed on a triangle on a cap), trunk (markers 4–6, fixed on a triangle on a trunk belt), and on the leg (see Figure 2). All markers were placed on the body side of the standing leg and were tracked by two video cameras (100 Hz). The video cameras were set up at a height of 1 m and were positioned perpendicular to each other with a focus on the center of the slackline. The cameras were spaced approximately 6 m apart and both were placed 6 m away from the participant. In this way, all markers remained in view of both cameras. Two flood lights were fixed directly below the cameras in order to improve marker reflection and hence tracking. The two cameras were synchronized and calibrated with a calibration frame. The method of Direct Linear Transformation (DLT) was used to obtain 3D data from the two 2D camera views.

#### ASL mobile eye system

The ASL Mobile Eye system (Applied Science Laboratories, Bedford MA, USA) is a compact eye tracking system. The eye tracking optics – mounted on a pair of lightweight goggles – consists of a scene camera, an infrared eye camera with built-in infrared diodes and an infrared mirror. The optics are unobtrusive and light (76g) and the recording device is small enough to be worn on a hip pack. The goggles are available in both children and adult sizes. Sampling rate is 30 Hz. Given its compact and lightweight design, the system allows the assessment of gaze behavior during natural tasks such as balancing on the slackline.

### Experimental procedure

All participants had three sessions on three separate days spaced 5–10 days apart. Sessions 1 and 2 were practice sessions, which were conducted in a gymnasium near participants’ homes. Session 3 was the measurement session and was conducted in a movement laboratory.

At the beginning of the first practice session, body weight, height, and foot area of participants was measured. Moreover, each participant was tested for his/her proprioception with a test described in Lord et al. (2003). In this test, participants sit on a chair with their knees aligned and attempt – without the aid of vision – to align their lower legs on either side of a vertical clear acrylic sheet. The difference, in degrees, between the orientations of both lower legs is subsequently recorded.

After these preparatory measurements, we provided slackline training, which started and ended with three trials in which the duration that participants could stand on the slackline was measured. The first time measurements at the very beginning of the training session are further referred to time at pre-test (*t*_pre_). In these test trials, participants were asked to balance on the slackline on their preferred leg for as long as possible. Moreover, participants were instructed to fixate their gaze on a smiley (diameter 11.5 cm) which was attached to the slackline anchor at approximately 2.5 m from participant’s eyes and at a downward angle of exactly 30° for all participants. Apart from the absence of the above-described preparatory measurements, practice session 2 was identical to practice session 1. The main rationale for the two practice sessions was to ensure that participants could stand on the slackline long enough to collect meaningful kinematic, kinetic, and gaze data in the subsequent measurement session. Besides exercises on one leg, which are in accordance with the pre- and post-test measurements, many exercises on two legs or involving walking were implemented to ensure a varied, motivating training. The two training sessions lasted 45 min each.

In the subsequent measurement session, after a short training practice of 15 min, participants were equipped with 25 reflective markers, as well as the mobile eye system (see Figure 2). They then assumed the “ready position” with their preferred leg on the slackline and their gaze directed at a LED light (the “nose” of the smiley) that was used to synchronize gaze, motion, and force measurements. Once the LED lit up, they were to start their attempt and keep their gaze fixed on the smiley as in all previous duration tests. The participant was considered to balance on the slackline from the moment the supporting foot left the ground (foot-off) until the moment of ground contact with any part of the body. Each participant performed three such trials, the longest of which was considered for further analysis. The following variables were measured: (1) time on the slackline, (2) body and slackline kinematics, (3) slackline reaction forces, and (4) gaze behavior.

### Data reduction

The first 0.2 and last 0.5 s of balancing were removed before motion and force data analysis to ensure the analysis of “steady state” balancing. Automatic tracking of the body markers allowed for position data of all markers at each time point. 2D coordinates of point of gaze of each frame were determined as follows. The middle of the smiley – the instructed fixation point – was taken as the origin of a vertical 2D (*x*, *y*) coordinate system. A large sheet with fine-grained visual structure (it contained the picture of a striped cat) was positioned directly behind the vertical plane through the fixation target and orthogonal to the horizontal slackline axis. It contained known reference lengths of both horizontal and vertical extent, to allow for a representation of gaze position in meters. For each sample of the balancing trial, the *x*- and *y*-coordinates (in meters) of gaze fixations in this plane were then determined from the cross-hair in the scene camera. These *x*-*y* time series served as an index of GM. Missing motion and gaze data were interpolated with a piecewise cubic interpolation for gaps no longer than 0.3 s. All data were then filtered with a second order low-pass Butterworth filter with a cut-off frequency of 10 Hz.

For the slackline dynamics, resultant forces for each sample were calculated from (1) forces measured at both ends of the slackline and (2) the movement of four slackline markers: two near the middle and two at the suspension points. As an index of PS, the interquartile range (IQR) of the angle between the vertical and the resultant force at each frame was determined. The rationale for this index is as follows. Perfect quiet stance on the slackline is characterized by a slackline reaction force pointing vertically upwards. Any disturbance of this stable quiet stance leads to rotations of the slackline reaction force away from vertical. The larger the disturbance of quiet stance the larger the deviations of the slackline reaction force from vertical. Hence, deviations of the slackline reaction force from vertical are equivalent with COP movement on a stable force plate: they both signify a disturbance of quiet stance.

Gaze stability was assessed by calculating the area of the 95% confidence ellipse of all measured gaze coordinates. Head-in-space, trunk-in-space and head-on-trunk stability were assessed by calculating head-in-space rotation (HR), trunk-in-space rotation (TR), and head-on-trunk rotation (HTR) as follows: $\sqrt{{\text{IQR}}_{\text{roll}}^{2}+{\text{IQR}}_{\text{yaw}}^{2}{\text{ + IQR}}_{\text{pitch}}^{2}}.$ For head stability, we additionally considered head-in-space translation: $\text{Ht = }\sqrt{x^{2}+y^{2}+z^{2}}$. These formulas were derived from the Pythagorean Theorem and define HR, TR, HTR, or Ht geometrically as the longest vector that fits within the volume spanned by the three orthogonal rotation/translation vectors. The trunk and HTR time series of two participants (one child and one adult) could not be analyzed due to occlusion of the trunk markers with the arms.

### Statistical analyses

As most of the data were not distributed normally, non-parametric statistics were adopted for all analyses. The 8-year-olds and adults were compared using a Mann–Whitney *U* test for the variables proprioception, time on the slackline at pre-test (*t*_pre_), time on the slackline at post-test (*t*_post_), GM, PS, HR, Ht, TR, and HTR. Furthermore, a cross-correlation of HTR and TR time series was performed. The cross-correlation coefficient was compared between groups using a Mann–Whitney *U* test. A Wilcoxon signed ranks test allowed for a comparison of *t*_pre_ and *t*_post_, head-in-space and head-on-trunk, and head-in-space and TRs within each age group. A planned set of bivariate Spearman correlation coefficients was calculated for each age group as well as across age groups: *t*_post_ – PS, *t*_post_ – GM, *t*_post_ – HR, *t*_post_ – Ht, *t*_post_ – TR, PS – GM, PS – HR, PS – TR, and GM – HR. The critical α level was set to 0.05 and no *p*-value adjustments for multiple comparisons were adopted. As a measure of effect size, *r* was calculated as $Z∕\sqrt{N}$ (where *Z* is the approximation of the observed difference in terms of the standard normal distribution and *N* is the total number of samples). *r* = 0.1 is considered small, *r* = 0.3 medium, and *r* = 0.5 a large effect size. See Table 2 for an overview of the abbreviations of the reported outcome measures.

**Table 2:** **Outcome measures**.

Measures	Abbreviation
Time on slackline at pre-test (s)	*t*_pre_
Time on slackline at post-test (s)	*t*_post_
Postural stability (°)	PS
Gaze movement (m^2^)	GM
Head-in-space rotation (°)	HR
Head-in-space translation (m)	Ht
Trunk-in-space rotation (°)	TR
Head-on-trunk rotation (°)	HTR
Cross-correlation between HR and TR (−)	CC HR-TR

## Results

Mann–Whitney *U* tests between 8-year-olds and adults revealed statistically significant differences for time on the slackline at post-test (*t*_post_), PS, head-in-space rotation (HR), head translation (Ht), GM, and proprioception measures. No statistically significant differences were found for the time on the slackline at pre-test (*t*_pre_), trunk-in-space rotation (TR), HTR, and the cross-correlation between HR and TR (see Table 3). The details of the analyses are described below.

**Table 3:** **Mann–Whitney *U* statistics between children and adults for proprioception, time on the slackline at pre-test (*t*_pre_) and at post-test (*t*_post_), postural stability (PS), gaze movement (GM), head-in-space rotation (HR), head-in-space translation (Ht), trunk-in-space rotation (TR), head-on-trunk rotation (HTR), and for cross-correlation between head-in-space and trunk-in-space rotations (CC HR-TR)**.

Measure	*N*	*U*	*Z*	*p*	*r*
Proprioception	31	42	−2.686	0.007	0.48
*t*_pre_	31	74	−1.310	0.201	0.24
*t*_post_	31	0	−4.453	<0.001	0.80
PS	21	0	−3.766	<0.001	0.82
GM	28	40	−2.397	0.016	0.45
HR	30	22	−3.432	<0.001	0.63
Ht	30	16	−3.696	<0.001	0.67
TR	27	49	−1.434	0.163	0.28
HTR	27	70	−0.566	0.596	0.11
CC HTR-TR	21	32	−1.268	0.224	0.28

*Effect size *r* was calculated according to $r=\frac{Z}{\sqrt{N}}$, with *N* the total number of samples*.

### Postural stability

**Figure 3:**
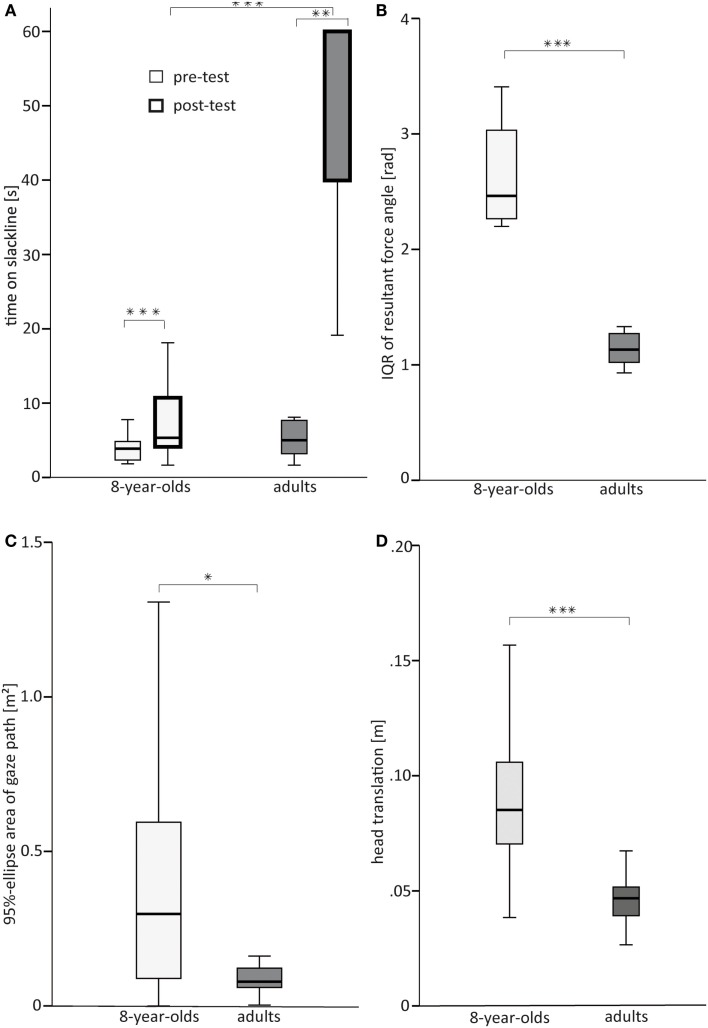
**Box plots featuring median, *Q*1 and *Q*3 with smallest and largest unbooked sample values shown as whiskers for (A) time on the slackline at pre- and post-test (B) interquartile range (IQR) of the resultant force angle (a larger value reflects lower postural stability), (C) 95% confidence ellipse area of gaze path, and (D) head translation**. *p < 0.05, ***p < 0.001.

At pre-test, time on the slackline did not differ significantly between groups (see Table 3). As revealed by Figure 3A, both groups balanced on the slackline for less than 5 s. At post-test, both groups increased their time significantly (8-year-olds: *Z* = −3.538, *p* < 0.001, *r* = 0.77; adults: *Z* = −2.803, *p* = 0.005, *r* = 0.89). At post-test, the adults were able to balance on the slackline much longer (49.8 ± 15.3 s, median: 60 s) than the 8-year-olds (7.3 ± 4.3 s, median: 5 s) (see Figure 3A).

Postural stability differed significantly between groups (see Table 3). Stability on the slackline was significantly lower in 8-year-olds than in adults (see Figure 3B).

The proprioception error score was significantly lower in the adult group (0.54° ± 0.43°, median 0.6°) than in the 8-year-olds (1.53° ± 1.27°, median 1.0°). The adults can thus be said to have a superior proprioception (in specific joint position sense) in this task than the 8-year-old participants.

### Gaze, head, and trunk movement

Gaze movement during balancing on the slackline was significantly larger in 8-year-olds than in adults (see Table 3): 8-year-olds let their gaze wander further away from the visual anchor point (see Figure 3C).

**Figure 4:**
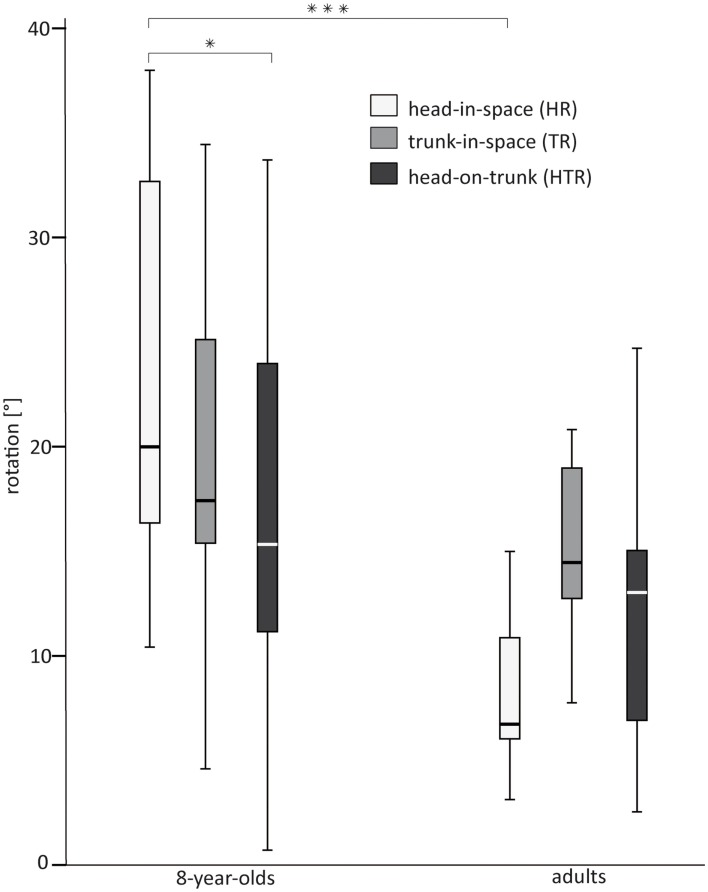
**Box plot featuring median, *Q*1 and *Q*3 with smallest and largest unbooked sample values shown as whiskers for head-in-space rotation (HR, left box), trunk-in-space rotation (TR, middle box), and head-on-trunk rotation (HTR, right box) in 8-year-old children and adults**. * for *p* < 0.05, *** for *p* < 0.001.

Both head-in-space rotation and head-in-space translation differed markedly between groups (see Table 3): both HR and Ht were larger in the 8-year-olds than in the adult participants (see Figure 3D and Figure 4). We found no such difference for TR and HTR (see Table 3 and Figure 4). The cross-correlation between head-on-trunk and TR was highly significant and negative in both 8-year-olds (median *r* = −0.73, *Q*_1_ = −0.95, *Q*_3_ = 0.02) and adults (median *r* = −0.93, *Q*_1_ = −0.97, *Q*_3_ = −0.76). The median correlation coefficients were not significantly different between groups (see Table 3).

For the 8-year-olds, HTR was significantly smaller than head-in-space rotation (*Z* = −2.417, *p* = 0.016, *r* = 0.57; see Figure 4). No difference between head-on-trunk and head-in-space rotation was found for the adults (*Z* = −0.415, *p* = 0.678, *r* = 0.14). The difference between head-in-space and TRs failed to reach significance, both in the 8-year-olds (*Z* = −1.459, *p* = 0.145, *r* = 0.34) and in the adults (*Z* = −1.400, *p* = 0.161, *r* = 0.49).

### Correlations

Table 4 summarizes the statistical results of the bivariate correlations between the different outcome measures. Only the correlation between *t*_post_ and HR was significant in both age groups. The high negative correlation in both groups indicates that longer balancing times on the slackline were associated with smaller head-in-space rotations in both 8-year-olds and adults. In the 8-year-olds, all other correlations were weak and non-significant. In the adults, HR and PS showed a strong significant correlation: smaller head-in-space rotations were associated with greater PS. The correlation between *t*_post_ and PS was also strong and significant in the adults: longer balancing times were associated with greater PS. Correlations across age groups were highly significant for the pairs HR – *t*_post_, PS – *t*_post_, and HR – PS (see Table 4) and significant for Ht – *t*_post_, and HR – GM. Larger head-in-space rotations were associated with shorter balancing times, lower PS, and larger GMs. Moreover, longer balancing times were associated with higher PS and smaller Hts.

**Table 4:** **Uncorrected bivariate correlations (Pearson)**.

Pair	Children			Adults			Over all		
	*r*	*p*	*N*	*r*	*p*	*N*	*r*	*p*	*N*
*t*_post_ – PS	0.232	0.446	13	−0.736	0.037	8	−0.838	<0.001	21
*t*_post_ – GM	−0.253	0.311	18	−0.425	0.221	10	−0.301	0.120	28
*t*_post_ – HR	−0.517	0.020	20	−0.729	0.017	10	−0.722	<0.001	30
*t*_post_ – Ht	0.134	0.573	21	−0.307	0.388	10	−0.552	0.002	30
*t*_post_ – TR	0.092	0.715	18	−0.289	0.487	8	−0.264	0.193	26
PS – GM	0.193	0.549	12	0.015	0.971	8	0.307	0.189	28
PS – HR	0.294	0.330	13	0.719	0.045	8	0.699	<0.001	30
PS – TR	0.228	0.500	11	0.344	0.504	6	0.260	0.314	17
GM – HR	0.318	0.198	18	0.841	0.002	10	0.391	0.040	30

**t*_pre_, Time on the slackline at pre-test; *t*_post_, time on the slackline at post-test; GM, gaze movement; PS, postural stability; HR, head-in-space rotation; Ht, head-in-space translation; TR, trunk-in-space rotation*.

## Discussion

In the present study, we aimed to assess the differences in PS, body kinematics and gaze behavior between 8-year-old children and young adults in the challenging, highly dynamic, and novel task of balancing on a slackline. We found marked differences in all these quantities. Below, we will first discuss the differences in PS between 8-year-olds and adults, and subsequently consider trunk, head, and gaze behavior as possible factors underlying this difference.

### Postural stability

Postural stability was inferred from the variability of the slackline reaction force. We argue that variations in the direction of this force during balancing on the slackline are conceptually equivalent to variations in the position of the COP during quiet standing on a force platform in that, in both cases, smaller variability is associated with greater PS. The high negative correlation we found between PS thus assessed and balancing time testifies to the validity of this argument. Our results thus show that the lower PS of children compared to adults during quiet standing generalizes to one-legged balancing on a slackline.

Unsurprising as this finding may be, it is interesting for two reasons. First, unlike PS differences observed during quiet standing, the current differences are not confounded by differences in task exposure between children and adults: although adults will inevitably have had more practice than children at the task of quiet standing, this is not the case for the task of balancing on a slackline. Second, if a balancing task does not critically challenge standing balance – as is the case in most balancing tasks adopted in the literature – differences in COP variability between children and adults may be attributed to a looser rather than worse postural control in children compared to adults. This means that the children might just sway more because the task allows them to, not because their lack of postural control forces them to. The present experimental task critically challenged standing balance in both children and adults and did not allow for a loosening of postural control so that an interpretation of reaction force variability in terms of postural control is unproblematic. Below we will discuss three factors that possibly underlie the worse postural control in children compared to adults: (1) the sensory systems and their integration, (2) anticipatory postural control, and (3) intersegmental coordination, particularly in relation to head-in-space stability.

Single-legged stance permits only a small amount of body sway given the small base of support. It therefore requires accurate and prompt sensory information (Riemann et al., 2003). Given that, in one-legged stance on the slackline, the base of support is not only small but also highly unstable, immaturity of the sensory systems is likely to put children at a crucial disadvantage in this task. Cumberworth et al. (2007), Hirabayashi and Iwasaki (1995), and Steindl et al. (2006) indeed suggest that the visual and vestibular systems reach adult-like capacity only around the age of 15–16 years. In the same studies it was suggested that somatosensory function is completely developed between 3 and 4 years. It must be noted though, that this latter suggestion is inconsistent with the results of the proprioception test (Lord et al., 2003) we included in the present experiment: the 8-year-old children’s performance was markedly inferior to that of the adults. If, however, we may still assume that in children the visual and vestibular systems are less mature than the somatosensory system, their postural control system is likely to rely more strongly on the latter. This was indeed shown by Peterka and Black (1990) for a variety of standing tasks. Riemann et al. (2003) suggested that, compared to two-legged standing, one-legged standing reduces the quality of somatosensory information. Moreover, Nashner and Peters (1990) noted that somatosensory information is degraded when standing on a compliant as compared to a non-compliant base of support. Given that our task involved one-legged standing on a compliant base of support, somatosensory information was likely to be difficult to utilize, which would have represented a further sensory disadvantage (in addition to the immaturity of the visual and vestibular systems) for the 8-year-olds in the present experimental task. In addition to the immaturity of the sensory modalities taken separately, their incomplete functional integration might represent a further factor underlying children’s inferior PS. Such an incomplete sensory integration in children has indeed been suggested (Woollacott et al., 1987; Peterson et al., 2006; Cuisinier et al., 2011). Given that the current experimental task involves multiple sensory systems and is highly challenging, a suboptimal sensory integration might indeed partly explain the relatively poor performance of our 8-year-olds.

The second factor possibly underlying children’s inferior performance on the slackline relates to the absence of anticipatory motor control (i.e., feedforward control) in children. Hatzitaki et al. (2002) found that children aged 11–13 years predominately used feedback control while quietly standing on one leg. Yet they predominantly engaged in feedforward control in a dynamic task in which the non-supporting limb was moved in the frontal and sagittal planes. We can thus assume that the participants in the present study needed well-developed feedforward control processes to successfully balance on the slackline. As suggested by Fujiwara et al. (2011) anticipatory postural control starts to develop from approximately 5–6 years of age and has not yet reached adult-like levels at age 11–12 years. Thus, the 8-year-olds in our study might have been less capable of using anticipatory postural adjustments than the adults, resulting in inferior performance on the slackline. To shed light on this issue, future studies should analyze (anticipatory) muscle activity in the present experimental paradigm.

Finally, the inferior postural control in children as compared to adults might be due to an immature intersegmental coordination, leading to a lack of head-in-space stabilization. Excessive head-in-space movement has been shown during posturokinetic activities (Assaiante et al., 2005) and during quiet standing with and without a visual dual task (Schärli et al., 2012). The present results on head, trunk, and GMs provide further support for this idea. It is to these results that we will turn now.

### Head, trunk, and gaze movement

Head stabilization in space seems a crucial determinant of postural and locomotor stability. As stated by Pozzo et al. (1990), the head is a natural frame of reference for action since it contains the two most important perceptual systems for detection of self-motion relative to space, the visual and vestibular systems. Balancing on a slackline constitutes a complex equilibrium task. It requires the active coordination of many segments, whereby head position could be used as a stable frame of reference from which dynamic equilibrium is organized as suggested by Pozzo et al. (1995). Their finding that falls in darkness were systematically preceded by head angular displacement out of its normal range supported this suggestion.

Our results demonstrate that head stability in space relates to prolonged stance duration and greater PS on the slackline. Eight-year-olds were clearly less proficient in stabilizing their heads in space as compared to the adult participants. Interestingly, however, rotations of the trunk did not differ between groups. The larger head-in-space rotations and translations found in 8-year-olds are therefore not caused by larger movements of the trunk. This finding supports the idea that children’s reduced balance ability is, in part, caused by their inability to stabilize the head-in-space. Assaiante and Amblard (1993) found that children younger than 7 years old adopted a head-stabilization-on-trunk-strategy (HSTS) and children older than 7 years and adults, a head-stabilization-in-space-strategy (HSSS) during stance and locomotion. Our 8-year-old participants seemed to do neither: both head-in-space and HTRs were substantial in this age group (as opposed to the adults who clearly adopted a HSSS as found by Assaiante and Amblard (1993). We will first discuss two possible reasons for the absence of a HSTS in our study, and subsequently provide a possible explanation of the absence of a HSSS.

First, a possible explanation for the absence of a HSTS is that our children participants were simply at a developmental stage at which the head does not move with the trunk anymore. Another explanation is that, in highly challenging tasks like slacklining, a HSTS is not viable. Indeed, with the large trunk-in-space rotations observed in both age groups, it seems to pose an unnecessary challenge to the visual and vestibular systems to rotate the head with the trunk. Rather, it seems crucial to counter-rotate the head relative to the trunk in order to maintain a stable frame of reference for these two systems (cf. Pozzo et al., 1990). A main determinant of a stable frame of reference for the visual system seems to be a horizontal orientation of the interorbital axis (Mouchnino et al., 1992). The high negative correlations between head-on-trunk and TRs in both age groups may thus point to attempts to maintain a horizontal interorbital axis. Our 8-year-olds and adults both seemed to perform such counter-rotations, even though the 8-year-olds clearly failed to thus achieve head-in-space stability. It follows that our children may have indeed opted for a HSSS, yet failed to successfully implement it. Given that, on this view, negative correlations between head-on-trunk and trunk-in-space may be taken as a marker of a HSSS rather than a HSTS, it would be interesting to see whether they also occur in younger children under highly challenging balancing conditions. Unfortunately, the current slackline paradigm would prove too challenging for most children under 8 years old; another experimental task should be considered. The beam-balancing task of Pozzo et al. (1995) may be more suitable.

Remarkably, Guitton et al. (1986) found that the control processes underlying head stability are strictly dependant on voluntary control. These authors observed that head stability was much worse in a mental arithmetic task as compared to a task wherein the head had to be kept stable intentionally. Also, their results showed that the short-latency vestibulo-collic and cervico-collic reflexes are superseded by alternative long-latency visual and vestibular tracking mechanisms when head-in-space stability is required. These findings may provide an explanation of the current findings in the 8-year-olds. The children might have been less aware than the adults that head stabilization is crucial for successful balancing. Adults, on the other hand, might have purposefully adopted head-in-space stabilization because they knew this to be a crucial task requirement. A possible lack of such awareness in the 8-year-olds may thus have negatively influenced their performance. The finding that head stability can be trained (Morimoto et al., 2011) is in line with the idea that head stabilization requires conscious effort. Vaugoyeau et al. (2008) suggest that the ability to stabilize the head-in-space does not develop without targeted training in tasks like standing or moving on irregular and/or slanted terrain. Given that the current slackline task would certainly constitute a proper training environment for the development of head-in-space stabilization, it might provide a means of accelerating children’s development of adult-like postural control. The finding of Morimoto et al. (2011) that gaze stability training involving voluntary head and eye movement and stabilization improved PS testifies to the feasibility of this approach.

Turning now to gaze stabilization, the 8-year-olds moved their gaze significantly more than the adult participants, even though all were advised to fixate a stationary target. Children thus achieved neither head nor gaze stability. The absence of gaze stability might point to an inability to use a visual “anchor” for PS. In support of this idea, Riach and Hayes (1987) proposed that children do not derive the same benefit from a stationary visual fixation point as adults do. They stated that this might be due either to a less effective fixation related to the distractibility and limited attention-span of children or to an inability to properly make use of the (retinal and extra-retinal) information granted by such fixation. Vickers (2009) supports the idea that attention deficits may negatively affect gaze stabilization: she emphasizes that – in athletes – gaze and attention shifts generally go together. Furthermore, it has been shown that a quiet eye (i.e., long fixations) is a clear sign of expertise in sports like golf and basketball (Vickers, 2009) and that it can be trained (Vine and Wilson, 2011; Vine et al., 2011). So, in addition to head stability, gaze stability during slacklining seems to be a sign of expertise and is obviously trainable. Both, a less effective fixation related to the distractibility and limited attention-span and an inability to properly make use of the (retinal and extra-retinal) information granted by such fixation, seem to apply in the children in our study: they did not fixate as effectively as adults, and showed no significant correlation between head and GM, which suggests that fixation did not help them achieve a stable reference frame. As Nashner (2009) pointed out, gaze is difficult to stabilize unless the body is also stable. Stoffregen et al. (2007) similarly claimed that a minimization of postural sway serves accurate visual fixations. The large body movements in the present experimental task would have certainly posed a challenge for the visual system. Gaze control and postural control may thus be interdependent: head and PS might facilitate a stable gaze and gaze stability might facilitate head and PS. With respect to the latter contingency, a stationary visual target has been shown to be most effective for PS when it is very near (i.e., less than 1 m). If the anchor point is farther away, its visual fixation may even become useless for postural stabilization (Edwards, 1946; Paulus et al., 1989; Kapoula and Le, 2006). It might thus be argued that our anchor point was too far away to have a meaningful stabilizing effect. It must be noted, however, that the translations of participants’ point-of-view – and hence the associated movement parallax – were much larger in the present study compared to most studies investigating optimal visual fixation distance. As a result, both the retinal and extra-retinal information about self-movement granted by a fixation point at 2 m distance may be much more useful in slacklining than it is in the commonly studied task of quiet standing.

### Effect of training

As discussed above, children were – after three training sessions – clearly less stable on the slackline as compared to adults. It is, however noteworthy that children and adults did not differ in their time on the slackline at their very first trial (i.e., pre-test). Both children and adult participants were unable to balance on the slackline. At the post-test, after three training sessions, adults were able to stand on the slackline more than six times longer than the children. This clearly means that the adult postural control system adapts more effectively to the unfamiliar and novel task of slacklining. Keller et al. (2012) assume that the lateral swinging observed at the start of the learning process has its origin in muscle stretch-reflex activation provoked by the initial sideward deflection of the slackline. The stretch-reflex activation strongly counteracts the initial deflection and the slackline overshoots to the other side: a fast side-to-side oscillation of the base of support results, which subsequently causes uncontrollable body sway and loss of balance. Keller et al. found significantly reduced Hoffmann (H)-reflexes (as an indicator for enhanced presynaptic inhibition of Ia afferents) after slackline training. Reduced H-reflexes have been shown to go along with improvements in postural control (Taube et al., 2007a,b). Therefore, we assume that slackline training might have induced an enhanced presynaptic inhibition of Ia afferents to a larger extent in the adults than in the 8-year-olds. In addition to children’s immature gaze and head stabilization, this points to a further immaturity of children’s postural control systems.

In conclusion, children aged around 8 years are less stable in a highly challenging dynamic and novel balance task (i.e., slacklining) than young adults. Children’s limited ability to stabilize their gaze and maintain a horizontal interorbital axis (via head-in-space stabilization) seem to be crucial factors in their reduced PS on the slackline. On the one hand inefficient head stabilization provokes a quicker balance loss in a direct mechanical way; on the other hand it renders the sensory information from visual and vestibular systems more difficult to utilize. A failure to stabilize the head-in-space thus constitutes a double disadvantage in the slackline task and presumably in other balancing tasks as well. A training program specifically aimed at improving head-in-space and gaze stability may not only improve children’s performance in the specific task of slacklining. It may also accelerate the development of the postural control system as a whole. Future studies should test this hypothesis and if proven correct, specific (slackline-based) head and gaze stability training could be implemented in elementary school physical education programs.

## Conflict of Interest Statement

The authors declare that the research was conducted in the absence of any commercial or financial relationships that could be construed as a potential conflict of interest.
